# An Aggressive Neoplasm with Mixed Epithelial and Sarcoma-like Features, of Uncertain Primary Origin, Presenting as an Upper Lip Mass: A Case Report

**DOI:** 10.3390/jcm15114331

**Published:** 2026-06-03

**Authors:** Ha Eun Park, Chongsoo Park

**Affiliations:** 1 Graduate School of Medicine, Konkuk University, Seoul 05030, Republic of Korea; haslinhaeun@gmail.com; 2 Department of Plastic and Reconstructive Surgery, Pusan National University Hospital, School of Medicine, Pusan National University, Busan 49241, Republic of Korea

**Keywords:** carcinosarcoma, lip mass, epithelial-mesenchymal transition (EMT), metastatic sarcoma, high-grade tumor, cancer of unknown primary (CUP)

## Abstract

Carcinosarcoma is a rare and aggressive malignancy characterized by both epithelial and mesenchymal components. It most commonly arises in the uterus, lung, or gastrointestinal tract, whereas occurrence in the oral cavity is exceptionally rare. Here, we report a rare case of an aggressive neoplasm with mixed epithelial and sarcoma-like features that initially presented as a benign-appearing upper lip lesion. A 47-year-old male first presented in March 2025 with a painless upper lip mass that had persisted for two months. The lesion appeared clinically benign, and an excisional biopsy was performed. However, during surgery, intraoperative frozen section analysis revealed features highly suspicious for malignancy. Following surgery, systemic imaging demonstrated multiple hypermetabolic lesions in the lung, colon, liver, pancreas, adrenal glands, and lymph nodes. Biopsies from the lip, colon, and lung revealed a high-grade malignant tumor with variable epithelioid and sarcomatoid features across sampled sites. Immunohistochemistry showed co-expression of cytokeratin and vimentin, supporting mixed epithelial and sarcoma-like features. As no primary tumor was identified despite extensive work-up, the available findings favored an aggressive malignant neoplasm of uncertain primary origin with mixed epithelial and sarcoma-like features, although the final histologic sub-classification remained uncertain due to incomplete original pathology information. The patient subsequently received palliative systemic chemotherapy with an alectinib-based targeted regimen starting in April 2025, but showed progressive disease on follow-up, even with additional second-line gemcitabine/cisplatin and third-line pembrolizumab therapy. The patient ultimately succumbed in September 2025. This case highlights that even relatively subtle-appearing lesions require a high index of suspicion for malignancy, emphasizing the importance of early biopsy and comprehensive systemic evaluation. Carcinosarcoma, though rare, should be considered in the differential diagnosis of aggressive tumors arising in atypical locations.

## 1. Introduction

Carcinosarcoma is an extremely rare, high-grade malignancy, accounting for less than 1% of all tumors, and is characterized by both epithelial and mesenchymal differentiation [1,2]. It is now classified as a subtype of epithelial carcinoma containing high-grade carcinomatous and sarcomatous components, rather than a true mixed tumor [3]. These tumors display aggressive biological behavior with a strong tendency for local invasion and distant metastasis [4]. Histologically, EMT features may be suggested by co-expression of epithelial markers cytokeratin (CK) and mesenchymal markers (vimentin) on immunohistochemical (IHC) staining [5]. In cases where the primary site cannot be identified despite extensive evaluation, such malignancies are categorized as CUP, a heterogeneous group of metastatic tumors with no detectable origin at presentation [6].

Carcinosarcomas more commonly arise in the uterus, lung, or gastrointestinal tract, involvement of the oral cavity, particularly the lip, is exceedingly rare. To the best of our knowledge, the incidence or prevalence of cases presenting in the lip without an identifiable primary site has not been established in the literature [7,8,9,10]. In these atypical sites, it may present with relatively indolent clinical features, such as a painless and slow-growing mass, leading to potential delays in diagnosis and management [11].

Because of their rarity and deceptively benign appearance, such tumors may be misdiagnosed or overlooked. Clinicians should maintain a high index of suspicion for malignancy when evaluating persistent, atypical, or rapidly enlarging lip lesions—even though most lesions in this region, such as mucoceles, fibromas, and hemangiomas, are benign [12].

We present a rare case of a patient with a persistent upper lip mass that was initially considered benign but was ultimately found to be associated with an aggressive neoplasm with mixed epithelial and sarcoma-like features, of uncertain primary origin, involving multiple organs. This case highlights the importance of prompt biopsy, detailed histopathologic assessment, and comprehensive systemic evaluation in the management of atypical oral lesions. For the accuracy, transparency, and usefulness of case reports, the CARE checklist has been reported in Supplementary Materials.

## 2. Case Report

**Figure 1:**
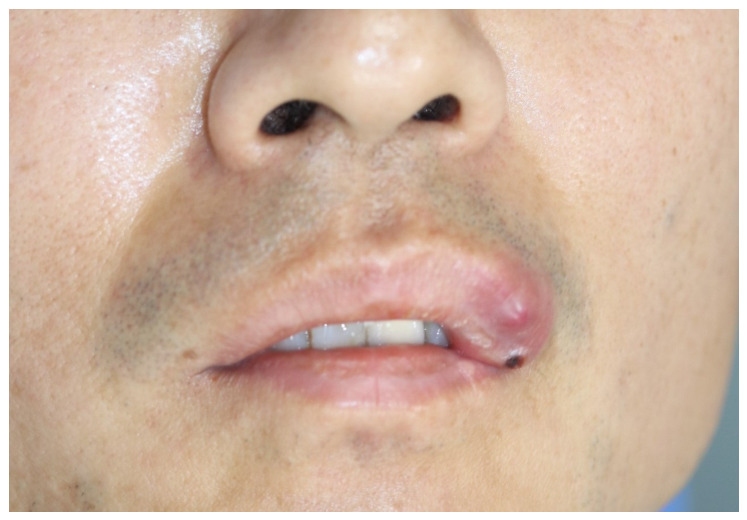
Initial clinical presentation of the left upper lip lesion. The lesion appeared as a firm, submucosal nodule of the upper lip. Fine-needle aspiration was attempted but yielded no material.

A 47-year-old male with no significant past medical history presented to our clinic with a two-month history of a painless, non-healing mass on the left upper lip (Figure 1). The lesion was firm, mobile, and measured approximately 15 mm in diameter, with overlying mucosa appearing intact and of normal color. No regional lymphadenopathy was noted. A detailed oral cavity examination was performed, revealing no additional lesions, ulceration, bleeding, or constitutional symptoms. The lesion was clinically considered benign, most likely a mucocele or fibroma. Fine-needle aspiration was attempted but yielded no material.

**Figure 2:**
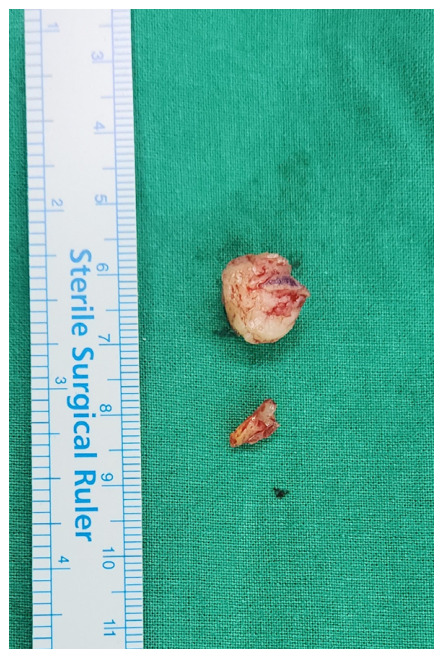
Intraoperative findings following excisional biopsy of the upper lip mass (1.1 × 0.9 cm). The mass appeared firmly adherent to the surrounding tissues. Gross examination revealed a firm, tan-white, heterogeneous cut surface suspicious for malignancy; definitive microscopic features were identified on frozen section analysis.

The patient underwent excisional biopsy under local anesthesia. Intraoperative frozen section analysis unexpectedly revealed features suggestive of malignancy (Figure 2). Same-day discharge was initially planned as per the routine excisional procedure at the day surgery center. However, due to the unexpected finding of a lesion highly suspicious of malignancy, discharge was postponed. The patient was admitted for further evaluation, and a multidisciplinary approach was undertaken to assess for possible metastatic disease.

Gross examination of the lip mass revealed a firm, mobile, submucosal nodule located in the upper lip, measuring approximately 1.5 × 1.3 × 1.0 cm. The overlying mucosa appeared intact without ulceration or discoloration. The lesion was well-circumscribed but extended into the submucosal and superficial muscular layers. Excisional biopsy was performed rather than needle sampling to allow full histologic evaluation. Microscopy showed a high-grade malignant neoplasm. In the representative H&E sections, the lip lesion showed predominantly epithelioid malignant cells, whereas spindle-shaped sarcomatoid morphology was more evident in the pulmonary lesion. Together with the immunohistochemical evidence of epithelial and mesenchymal differentiation, the overall findings favored a neoplasm with mixed epithelial and sarcoma-like features.

However, the original full pathology report and complete laboratory/pathology details were not available for review. Therefore, although the available morphologic and immunophenotypic findings favored a neoplasm with mixed epithelial and sarcoma-like features, the full pathological basis for this interpretation could not be confirmed. Accordingly, the final sub-classification should be interpreted with caution and remains subject to diagnostic uncertainty.

Following excisional biopsy of the upper lip lesion, contrast-enhanced CT (CECT) scans of the head and neck, chest, and abdomen were performed on 6 March 2025. The next day (7 March 2025), a bronchoscopy was conducted, revealing findings suspicious for multiple metastases. Subsequently, pancreatic MRI was obtained on March 8, followed by colonoscopy and whole-body Positron Emission Tomography–Computed Tomography (PET-CT) on 10 March 2025, which confirmed widespread metastatic involvement.

**Figure 3:**
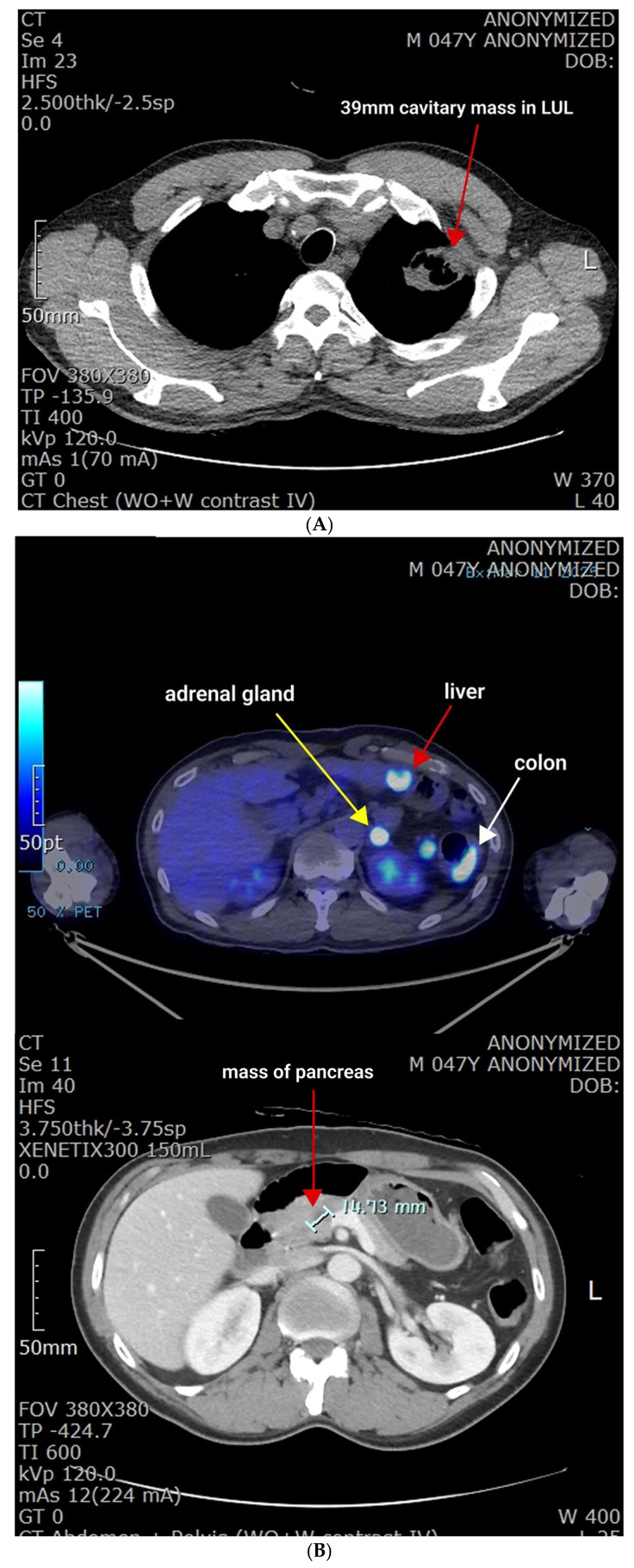

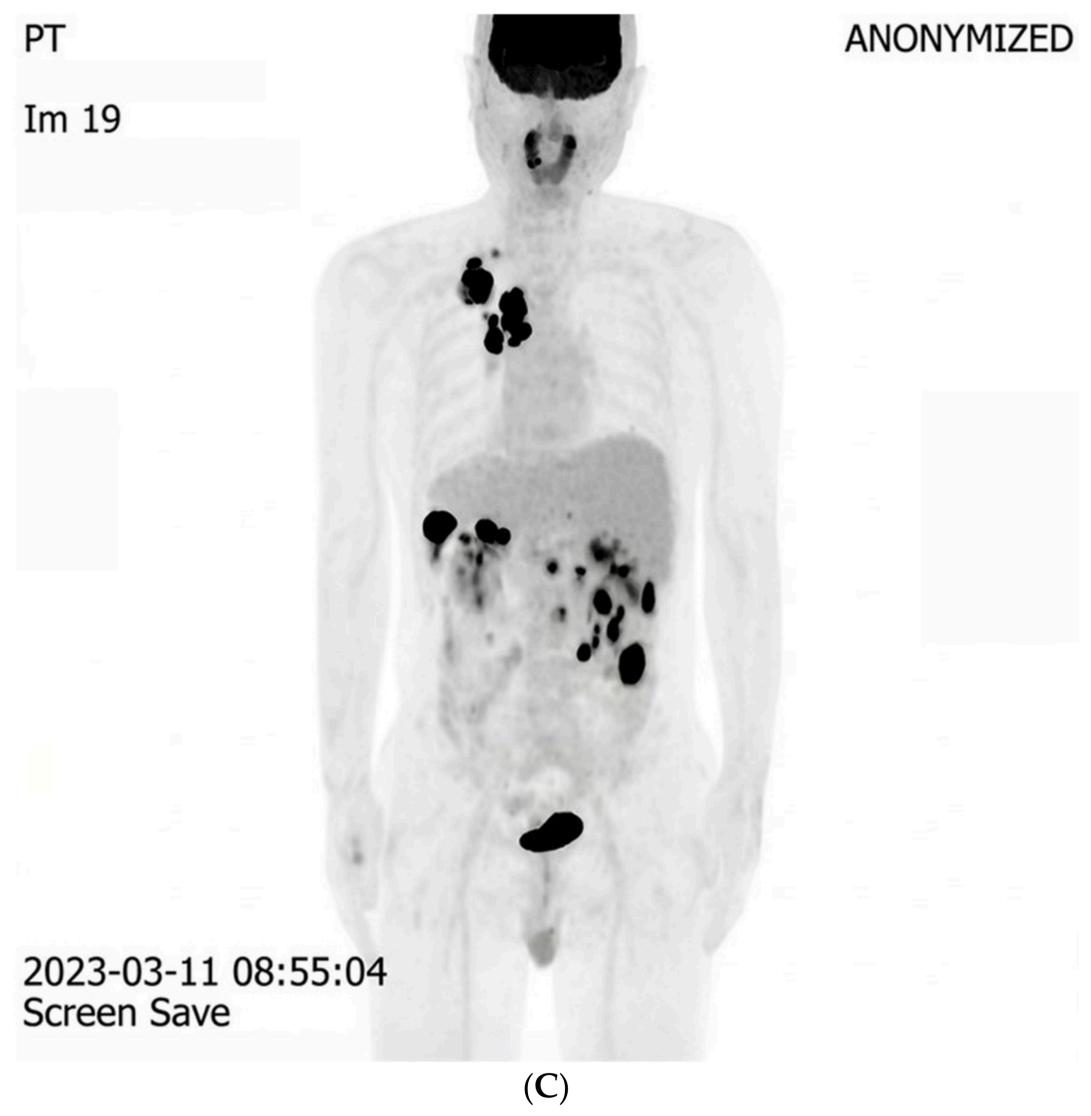
Cross-sectional imaging demonstrating widespread metastatic disease. (**A**) 39 mm cavitary mass in the left upper lobe (LUL). (**B**) Abdominal PET-CT (upper) and contrast-enhanced CT (CECT, lower) revealing metastatic lesions in the colon (upper image, white arrow; splenic flexure and descending colon), liver (upper image, red arrow; 2.5 cm, segment 3), pancreas (lower image, red arrow; 1.5 cm, neck), and left adrenal gland (upper image, yellow arrow; 1.6 cm). (**C**) PET-CT scan favoring systemic metastases across multiple organs.

A comprehensive systemic evaluation was initiated. CECT and MRI revealed multiple enhancing lesions in the lungs, liver, colon, pancreas, adrenal glands, and lymph nodes. PET-CT confirmed these findings, revealing multiple hypermetabolic foci with markedly increased uptake of fludeoxyglucose F18 (^18^F-FDG). The major lesions and their SUVmax values are summarized in Table 1. Mean reference SUVs were 1.7 in the mediastinum and 2.4 in the liver. PET-CT was performed after 6 h of fasting with intravenous administration of 5.2 MBq/kg ^18^F-FDG, and CT contrast enhancement used 100 mL of non-ionic iodinated medium (iohexol, 300 mg I/mL) (Figure 3).

**Figure 4:**
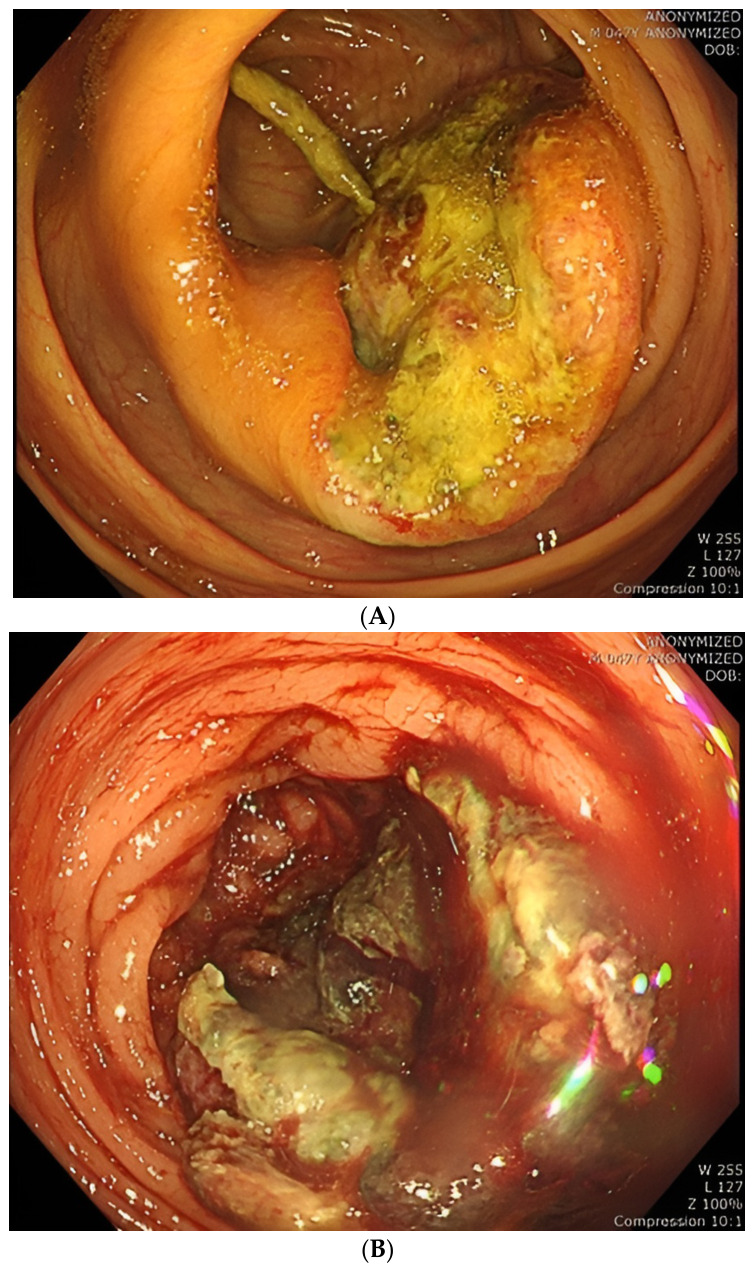
Colonoscopy findings of an aggressive neoplasm involving the colon. (**A**) Colonoscopy revealed a large, fungating, ulcero-infiltrative mass with yellowish exudate in the cecum. (**B**) Another ulcero-infiltrative mass with hemorrhagic and necrotic surface was noted in the descending colon.

Colonoscopy was performed to evaluate suspected colonic involvement. Endoscopic examination revealed a large, fungating, ulceroinfiltrative mass with yellowish exudate in the cecum, along with irregular, friable mucosal lesions extending to the descending colon. The cecal lesion appeared as an ulcerated, polypoid mass with mucosal edema and contact bleeding, while the descending colon showed circumferential infiltrative thickening with loss of the normal vascular pattern (Figure 4).

Targeted biopsies were obtained from both sites. Histopathologic examination demonstrated diffuse infiltration of atypical pleomorphic cells within the lamina propria and submucosa, showing marked nuclear atypia, prominent nucleoli, and high mitotic activity. IHC analysis revealed co-expression of CK and vimentin, supporting a mixed epithelial and mesenchymal immunophenotype in an aggressive neoplasm with mixed epithelial and sarcoma-like features.

Core needle biopsies were obtained from the dominant left upper lobe lung lesion, which had been identified on chest CECT, and from the colonic mass. Histopathologic examination of the sampled lesions showed overlapping high-grade malignant features. The lip and colonic lesions demonstrated predominantly epithelioid malignant cells, while the pulmonary lesion showed spindle-shaped sarcomatoid morphology. These findings, together with the immunohistochemical profile, supported a neoplasm with mixed epithelial and sarcoma-like features.

**Figure 5:**
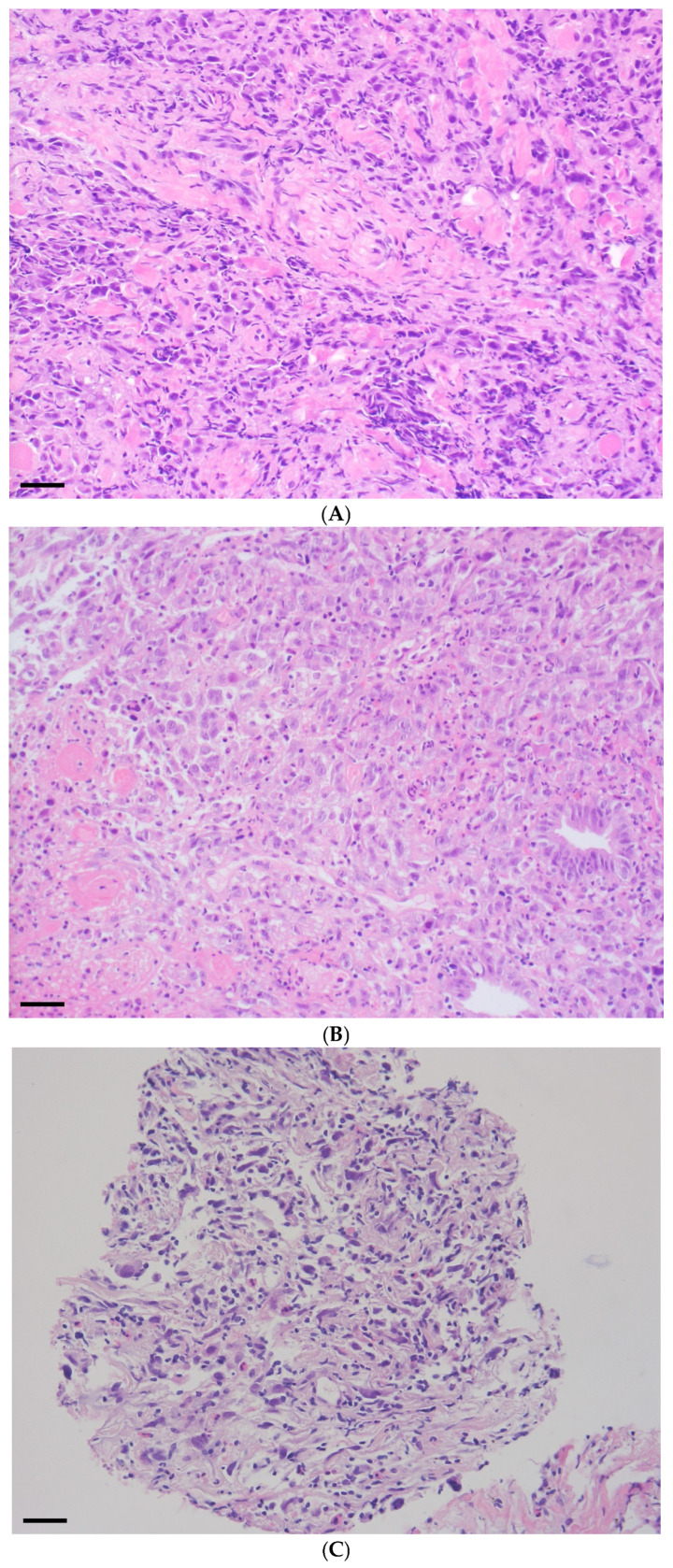
Histopathological features of the sampled lesions (H&E, ×200; scale bar = 100 μm). (**A**) The lip lesion shows malignant tumor cells with predominantly epithelioid morphology. Some cells show a crush artifact, which may obscure detailed cytologic assessment. (**B**) The colonic lesion similarly demonstrates malignant cells with predominantly epithelioid morphology. (**C**) The pulmonary lesion shows malignant spindle-shaped cells with sarcomatoid morphology.

Permanent hematoxylin and eosin (H&E) sections demonstrated a high-grade spindle cell neoplasm composed of pleomorphic tumor cells arranged in fascicles with marked nuclear atypia and brisk mitotic activity, averaging 22 mitoses per 10 high-power fields (HPF). Areas of geographic necrosis involving approximately 15% of the tumor volume were identified. Lymphovascular invasion (LVI) was present, confirmed by endothelial-lined vascular permeation on serial sections. The surgical margins were free of tumor, with the closest deep margin measuring 1.2 mm from the invasive front. These findings supported a high-grade malignant neoplasm with mixed epithelial and sarcoma-like features, raising the possibility of carcinosarcoma in the differential diagnosis (Figure 5).

**Figure 6:**
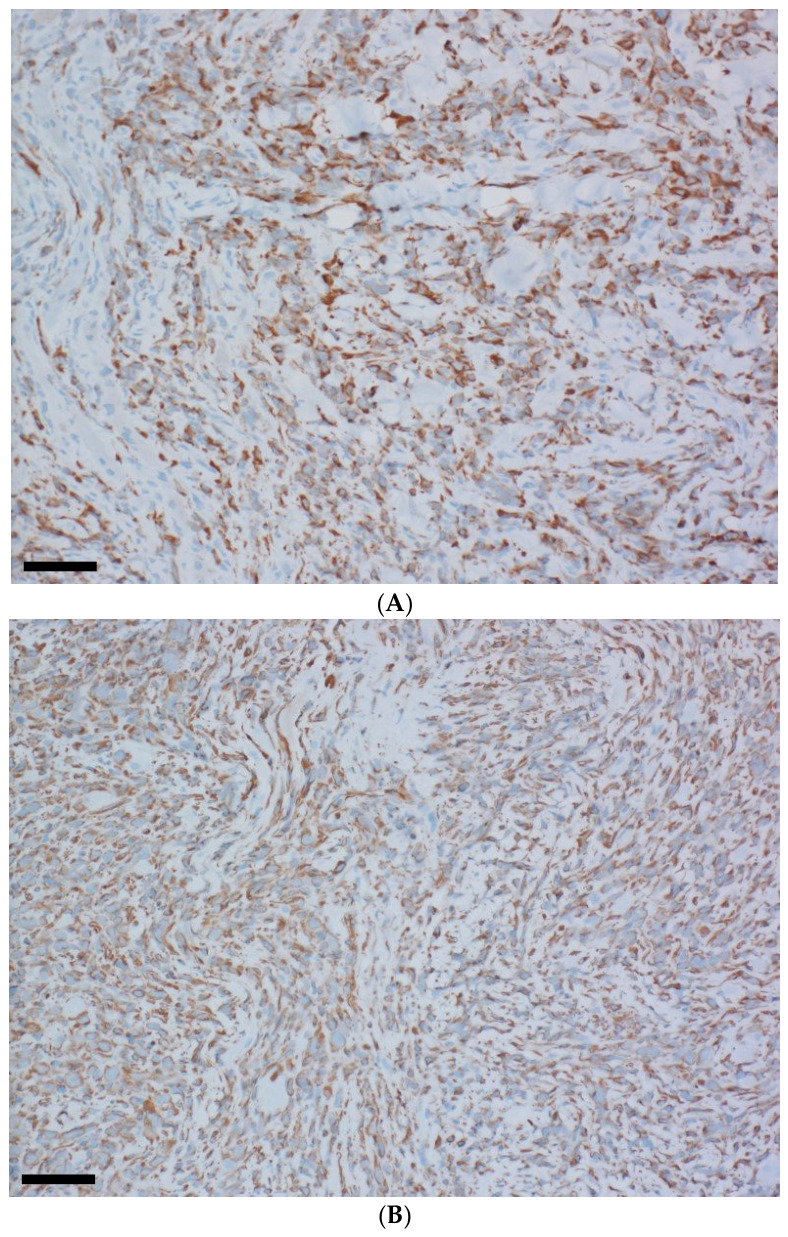

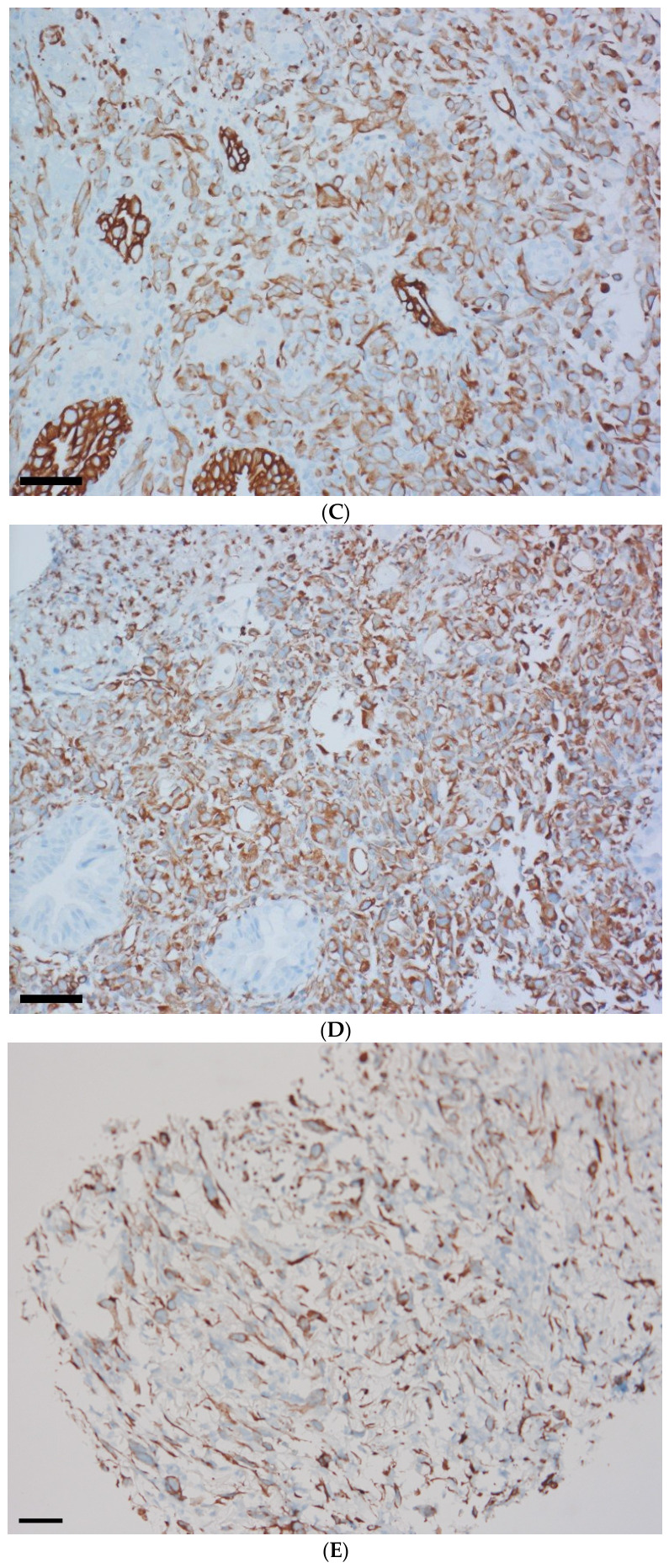
Immunohistochemical staining (IHC, ×200; scale bar = 100 μm) of the sampled lesions. Representative images show similar epithelial and mesenchymal marker expression patterns across the examined sites. (**A**) Cytokeratin IHC of the lip mass. (**B**) Vimentin IHC of the lip mass. (**C**) Cytokeratin IHC of the colonic lesion. (**D**) Vimentin IHC of the colonic lesion. (**E**) Cytokeratin IHC of the pulmonary lesion.

IHC staining demonstrated co-expression of CK and vimentin, supporting a mixed epithelial and mesenchymal immunophenotype in this neoplasm (Figure 6). The differential diagnoses considered included sarcomatoid carcinoma, spindle cell squamous cell carcinoma (SCC), and malignant melanoma. According to the 5th edition of the WHO Classification of Head and Neck Tumours (2022), sarcomatoid carcinoma represents a poorly differentiated epithelial malignancy that may show focal or complete loss of CK expression, particularly in high-grade spindle cell areas [3]. However, in the present case, the available morphologic and immunophenotypic findings favored an aggressive malignant neoplasm with epithelioid and sarcomatoid features. Nevertheless, because the original full pathology diagnostic report and complete diagnostic reasoning were unavailable, this distinction remains subject to uncertainty, as summarized in Table 2. Spindle cell SCC was ruled out by negative p40 and CK5/6 expression, and malignant melanoma was excluded based on negative S-100, HMB-45, and Melan-A staining. Taken together, the combined histomorphologic and immunophenotypic findings favored an aggressive neoplasm with mixed epithelial and sarcoma-like features, although definitive sub-classification remained limited by incomplete pathology information.

Although this immunophenotypic overlap is uncommon, it has been reported in certain sarcomas and poorly differentiated carcinomas. To determine the possible site of origin, an extended IHC panel was performed on both the epithelial and mesenchymal components. The carcinomatous component showed focal positivity for CK (AE1/AE3) and epithelial membrane antigen (EMA), with TTF-1 positivity in a few cells and negativity for p40, CDX2, and CK20, making primary tumors of squamous, gastrointestinal, or definite pulmonary origin less likely. The mesenchymal spindle cell component was negative for smooth muscle actin (SMA), desmin, MyoD1, S100, SOX10, DOG1, and CD34, excluding myogenic, neural, and gastrointestinal stromal differentiation. Taken together, these histologic and immunophenotypic findings, as summarized in Table 3, favor an aggressive neoplasm with mixed epithelial and sarcoma-like features, of uncertain primary origin, showing both epithelial and mesenchymal differentiation without IHC evidence favoring a specific organ origin.

As described above, to investigate the possibility of a primary tumor, a comprehensive diagnostic work-up was performed. This included thorough head and neck, dermatologic, and oral cavity examinations, as well as CECT scans of the chest, abdomen, and pelvis. In addition, bronchoscopy, pancreatic MRI, colonoscopy, and whole-body PET-CT were conducted to identify potential primary lesions. Histopathologic examinations from multiple involving lesions—including the lip, colon, and lung—revealed similar mixed epithelial and sarcoma-like features without any evidence suggesting origin from a specific organ. Despite these extensive evaluations, no definitive primary tumor was identified.

Given the extensive metastatic burden and absence of a definitive primary site, the patient was referred to the hemato-oncology department and initiated on palliative systemic chemotherapy. The first-line regimen consisted of alectinib, administered from 1 April to 24 April 2025 (two cycles), as selected by the hemato-oncology team in a palliative setting. However, progressive disease (PD) was documented, accompanied by intestinal obstruction and enlargement of pulmonary and nodal metastases. The patient subsequently underwent segmental small-bowel resection on 30 April 2025 to relieve the obstruction.

Second-line palliative chemotherapy with gemcitabine and cisplatin (gem/cis) was initiated from 13 May to 24 June 2025 (two cycles), but interval imaging again revealed disease progression. Third-line therapy with pembrolizumab was administered from 7 July to 27 August 2025 (three cycles), as selected by the hemato-oncology team in a palliative setting; however, further progression was observed.

At the latest follow-up in September 2025, the disease had progressed radiographically despite multiple lines of therapy, with new and enlarging metastases in the liver, pancreas, adrenal glands, colon, and retroperitoneum, as well as worsening ascites and peritoneal carcinomatosis. Although some pulmonary lesions showed partial necrosis with minimal reduction in size, the overall clinical course was consistent with progressive disease. The patient ultimately died of the disease in September 2025, approximately six months after diagnosis and initiation of palliative therapy.

## 3. Discussion

### 3.1. Pathogenesis and Terminology

Carcinosarcoma-like malignancies have historically been described using various terms—pseudosarcoma, sarcomatoid carcinoma, and collision tumor—reflecting earlier uncertainty about their pathogenesis, as summarized in Table 4 [13,14]. These tumors were once believed to arise from dual origins or represent reactive stromal changes. However, the 5th edition of the WHO Classification of Head and Neck Tumors (2022) redefined carcinosarcoma as a high-grade epithelial malignancy containing both carcinomatous and sarcomatous components, supporting its classification as a subtype of epithelial carcinoma rather than a biphasic or mixed neoplasm [3].

Four major theories explain carcinosarcoma pathogenesis. The collision theory suggests that independent epithelial and mesenchymal tumors coexist. The composition theory interprets the mesenchymal component as a stromal reaction. The combination theory proposes origin from a single pluripotent stem cell with divergent differentiation. The currently favored conversion model posits that epithelial cells undergo sarcomatoid transformation via EMT [15]. Molecular analyses revealing identical Tumor protein 53 (TP53) and KRAS proto-oncogene, GTPase (KRAS) mutations in both components strongly support this model [16].

EMT-associated sarcomatoid transformation has been documented in several malignancies. Examples include lung adenocarcinoma progressing to sarcomatoid carcinoma and radiation-induced sarcomatoid changes in head and neck squamous cell carcinoma or basal cell carcinoma after hedgehog-inhibitor therapy [17].

### 3.2. Exceptionally Rare Presentation as a Lip Mass

Carcinosarcomas most commonly occur in the uterus, lung, and gastrointestinal tract, but have been reported in the larynx, salivary glands, skin, esophagus, pancreas, colon, and ovaries. Involvement of the oral or perioral region—especially the upper lip—is exceedingly rare. Oral metastases comprise approximately 1% of all oral malignancies, most frequently involving the gingiva or mandible [18]; thus, the lip is an unusual site of presentation.

In this case, no primary tumor was identified despite comprehensive work-up, supporting a diagnosis of CUP, which accounts for about 2.3–4.2% of all malignancies [19]. Sarcomatoid carcinoma of unknown primary (SCUP) represents a rare subtype of cancer of unknown primary (CUP), accounting for approximately 4% of all CUP cases in a single institutional series [14]. This suggests that SCUP may be underrecognized within the CUP spectrum.

A review of the MD Anderson Cancer Center CUP registry (2001–2017) identified 48 patients with SCUP [14]. To our knowledge, no prior report has described carcinosarcoma of unknown primary initially manifesting as a lip mass with subsequent systemic metastases, making this case exceptionally rare.

### 3.3. Comparative Histopathological and Biological Features of Carcinoma and Sarcoma

Carcinomas and sarcomas differ fundamentally in their cellular origin, molecular pathways, and routes of dissemination, which underlie their distinct clinical behaviors. Carcinomas arise from epithelial cells lining organs and glands, showing cohesive growth patterns, desmosome junctions, and expression of epithelial markers such as CK and EMA [20]. In contrast, sarcomas originate from mesenchymal cells—fibroblasts, myocytes, or vascular elements—and exhibit spindle or pleomorphic morphology with immunoreactivity for vimentin, desmin, or SMA, reflecting mesenchymal differentiation.

From a pathobiological perspective, the distinction between these two tumor classes is critical, as it determines metastatic tropism and therapeutic response. Carcinomas predominantly spread through lymphatic dissemination, resulting in early nodal involvement, whereas sarcomas spread mainly via the hematogenous route, often metastasizing to the lungs, liver, or bones. This fundamental difference stems from the epithelial adherence properties and basement membrane interaction in carcinomas versus the stromal invasiveness and vascular affinity of mesenchymal cells in sarcomas [21].

In the context of carcinosarcoma, epithelial and sarcomatoid phenotypes may coexist within the same neoplasm, although their relative prominence may vary among sampled sites and representative sections. Immunohistochemically, this dual differentiation manifests as co-expression of epithelial markers (CK, EMA) and mesenchymal markers (vimentin, occasionally SMA). This feature may reflect EMT-related phenotypic change, in which epithelial tumor cells acquire mesenchymal traits associated with increased migratory capacity and invasiveness [22]. Recognizing this hybrid immunophenotype is essential for distinguishing true carcinosarcoma from sarcomatoid carcinoma, spindle cell squamous cell carcinoma, or other mimickers.

### 3.4. Diagnostic Challenges and Clinical Implications

The present case involved a 47-year-old male with no significant medical history who presented with a painless upper lip mass. Clinically, it resembled benign lesions, leading to an initial plan for simple excision. However, intraoperative frozen section revealed malignancy, prompting further imaging and multidisciplinary management. After diagnosis, the patient reported mild indigestion and some weight loss but had attributed these symptoms to recent overwork and did not suspect malignancy. At the time of referral to the hemato-oncology department and initiation of treatment, the general condition was tolerable with an Eastern Cooperative Oncology Group Performance Status (ECOG PS) of 1, and only a mild cough was present.

The principal instructive value of this case lies in its clinical presentation rather than in definitive histologic sub-classification. A relatively young patient presented with a small, painless, benign-appearing upper lip mass, yet subsequent evaluation revealed a rapidly progressive systemic malignancy. This emphasizes that persistent or atypical oral and perioral lesions should be biopsied even when they appear clinically innocuous.

Delays in diagnosis and initiation of therapy can lead to disease progression and psychological distress. However, in this case, the malignancy exhibited an inherently aggressive course, progressing rapidly despite prompt diagnosis and initiation of systemic therapy. This case underscores the importance of maintaining vigilance and pursuing early biopsy and systemic evaluation of persistent oral lesions, even when they appear benign, to enable timely diagnosis in potentially aggressive tumors. This case also illustrates that, in selected patients with aggressive malignancy and uncertain histologic sub-classification, empiric systemic therapy may be considered in a palliative setting after multidisciplinary discussion. In the present case, systemic treatment was initiated despite diagnostic uncertainty because of the extensive metastatic burden and rapid clinical progression.

### 3.5. Limitations

This report has several limitations. Despite extensive evaluation, no primary tumor site was identified. Therefore, while the overall findings favored an aggressive neoplasm with mixed epithelial and sarcoma-like features, of uncertain primary origin, definitive classification as either metastatic carcinosarcoma or a primary lip-region carcinosarcoma was not possible. Immunohistochemical testing was limited to essential markers, and molecular analyses such as next-generation sequencing were not performed, restricting further assessment of tumor lineage and clonality. In addition, detailed methodological information, including specific antibody clones and dilutions, could not be retrieved, which limits reproducibility. These factors reduce the certainty of conclusions regarding the tumor’s precise origin, histogenesis, and biological behavior. Although the available histomorphologic and immunophenotypic findings supported mixed epithelial and sarcoma-like features, the final sub-classification should be interpreted with caution.

## 4. Conclusions

This case highlights the clinical importance of maintaining suspicion for malignancy when evaluating persistent, benign-appearing upper lip lesions, even in relatively young patients. Although the available findings supported mixed epithelial and sarcoma-like features, the final histologic sub-classification remained uncertain. The main lesson to be learned from this case is that an apparently innocuous oral lesion may represent the first manifestation of an aggressive systemic malignancy, requiring prompt biopsy, comprehensive systemic evaluation, multidisciplinary discussion, and, when appropriate, empiric palliative systemic therapy.

## Figures and Tables

**Table 1:** Summary of major hypermetabolic lesions on PET-CT.

Site/Lesion	SUVmax	Size/Additional Findings
Left upper lobe cavitary mass	24.4	3.7 cm; anterior chest wall invasion
Left hepatic lobe lesion	16.7	2.5 cm
Left adrenal gland lesion	26.5	Not specified
Right adrenal gland lesion	5.3	1.6 cm
Pancreatic neck lesion	10.8	1.5 cm
Descending colon lesion	26.9	Not specified
Cecal lesion	23.9	Not specified
Ileal lesion	16.1	Not specified
Left supraclavicular lymph node	18.0	Hypermetabolic lymphadenopathy
Prevascular/paraaortic/subaortic lymph nodes	23.6	Hypermetabolic lymphadenopathy
Paratracheal/hilar lymph nodes	18.5	Hypermetabolic lymphadenopathy
Left upper lobar lymph node	25.5	Hypermetabolic lymphadenopathy
Superior mesenteric lymph node	8.8	Hypermetabolic lymphadenopathy
Ileocolic lymph node	19.1	Hypermetabolic lymphadenopathy
Left colic branch lymph node	9.0	Hypermetabolic lymphadenopathy
Left upper lip superficial lesion	12.2	Hypermetabolic superficial lesion
Bilateral palatine tonsils	10.5	Increased uptake
Neck level I/II and right level V nodes	up to 3.4	Mild uptake

Note. Mean reference SUVs were 1.7 in the mediastinum and 2.4 in the liver. PET-CT was performed after 6 h of fasting following intravenous administration of 5.2 MBq/kg 18F-FDG. Contrast-enhanced CT was obtained using 100 mL of non-ionic iodinated contrast medium (iohexol, 300 mg I/mL).

**Table 2:** Immunohistochemical profile and differential diagnostic considerations.

Differential Diagnosis	Key IHC Markers	Findings in This Case	Interpretation
Sarcomatoid carcinoma	Cytokeratin (+), Vimentin (focal +), EMA (+)	Cytokeratin and vimentin diffusely (+)	True biphasic pattern supports carcinosarcoma rather than sarcomatoid carcinoma
Spindle cell SCC	p40 (+), CK5/6 (+)	Both negative	Rules out squamous epithelial origin
Malignant melanoma	S-100 (+), HMB-45 (+), Melan-A (+)	All negative	Excludes melanocytic neoplasm
Carcinosarcoma	Cytokeratin (+), Vimentin (+)	Both diffuse (+)	Consistent with epithelial–mesenchymal differentiation

**Table 3:** Extended immunohistochemical profile for determination of primary site.

Marker	Component Tested	Result	Interpretation/Excluded Origin
Cytokeratin (AE1/AE3)	Carcinomatous	Positive	Supports epithelial differentiation
EMA	Carcinomatous	Positive	Supports epithelial origin
p40/p63	Carcinomatous	Negative	Argues against squamous differentiation
CD34	Mesenchymal	Negative	Argues against vascular or GIST lineage, Supports non-vascular origin
CD56	Neural or Neuroendocrine	Focal positive	Suggests possible but non-specific neuroendocrine differentiation
Vimentin	Mesenchymal	Positive	Supports mesenchymal component
SMA	Mesenchymal	Negative	Does not support myogenic differentiation
Desmin	Mesenchymal	Negative	Rules out smooth muscle tumor
Ki-67	Tumor proliferation index	High (about 50%)	Indicates high proliferative activity, consistent with aggressive tumor behavior.

**Table 4:** Synonyms and variant terminologies used to describe carcinosarcoma in the literature.

Carcinosarcoma
Bizarre squamous cell carcinoma
Carcino(pseudo)sarcoma
Carcinoma with pseudosarcoma
High-grade epithelioid neoplasm
High-grade or poorly differentiated carcinoma
Malignant neoplasm favor sarcoma
Malignant spindle cell and epithelioid neoplasm
Metaplastic carcinoma
Pleomorphic carcinoma
Pleomorphic sarcomatoid malignant neoplasm
Polypoid squamous cell carcinoma
Poorly differentiated carcinoma with sarcomatoid features
Pseudocarcinoma
Pseudocarcinosarcoma
Pseudosarcoma
Pseudosarcomatous carcinoma
Sarcomatoid carcinoma
Sarcomatoid malignant neoplasm
Sarcomatoid malignant neoplasm favor anaplastic carcinoma
Spindle cell (sarcomatoid) carcinoma
Spindle cell carcinoma
Spindle cell variant of squamous carcinoma
Squamous cell carcinoma with pseudosarcoma
Squamous cell carcinoma with sarcoma-like stroma

## Data Availability

Data is contained within the article.

## Supplementary Materials

The following supporting information can be downloaded at: https://www.mdpi.com/article/10.3390/jcm15114331/s1, Table S1: The CARE Checklist for Case Report.

## Abbreviations

The following abbreviations are used in this manuscript:

EMT	Epithelial–mesenchymal transition
CUP	Cancer of Unknown Primary
CK	Cytokeratin
IHC	Immunohistochemical
PET-CT	Positron Emission Tomography–Computed Tomography
^18^F-FDG	Fludeoxyglucose F18
SUV_max	Standardized Uptake Value maximum
H&E	Hematoxylin and Eosin staining
HPF	High-power field
LVI	Lymphovascular invasion
SCC	Squamous cell carcinoma
EMA	Epithelial membrane antigen
SMA	Smooth muscle actin
PD	Progressive disease
SCUP	Sarcomatoid carcinoma of unknown primary
TP53	Tumor protein 53
KRAS	KRAS proto-oncogene, GTPase
ECOG PS	Eastern cooperative oncology group performance status
CECT	Contrast-enhanced CT
