# Supplementary material for: An Aggressive Neoplasm with Mixed Epithelial and Sarcoma-like Features, of Uncertain Primary Origin, Presenting as an Upper Lip Mass: A Case Report
Source: J Clin Med. 2026 Jun 3;15(11):4331. doi: 10.3390/jcm15114331 (PMC13258668; doi:10.3390/jcm15114331)
Supplement: Supplementary file 1 [file jcm-15-04331-s001.zip › jcm-4310827-supplementary.pdf]

Supplementary Material S1. CARE Checklist

Manuscript title: Metastatic Carcinosarcoma of Unknown Primary Origin Presenting as an Upper Lip Mass: A Case Report

Topic	Item	Checklist item description	Reported on page/line	Remarks
Title	1	The diagnosis or intervention of primary focus followed by the words “case report”	p. 1, lines 1–3	Reported
Key Words	2	Two to five key words that identify diagnoses or interventions in this case report, including “case report”	p. 1, lines 40	Reported; “case report” added to the keyword list
Abstract	3a	Introduction: What is unique about this case and what does it add to the scientific literature?	p. 1, lines 12–14	Reported
Abstract	3b	Main symptoms and/or important clinical findings	p. 1, lines 16–18	Reported
Abstract	3c	The main diagnoses, therapeutic interventions, and outcomes	p. 1, lines 19–25	Reported
Abstract	3d	Conclusion: What is the main take-away lesson from this case?	p. 1, lines 31–33	Reported
Introduction	4	One or two paragraphs summarizing why this case is unique, with references if appropriate	p. 2, line 52 -62	Reported
Patient Information	5a	De-identified patient-specific information	p. 2, line 70	Reported
Patient Information	5b	Primary concerns and symptoms of the patient	p. 2, lines 70–76	Reported
Patient Information	5c	Medical, family, and psycho-social history including relevant genetic information	p. 2, lines 70–76	Partly reported; no relevant family/genetic history was available in the manuscript
Patient Information	5d	Relevant past interventions with outcomes	Not applicable	No relevant prior intervention before presentation
Clinical Findings	6	Significant physical examination and important clinical findings	p. 2, lines 77–83	Reported
Timeline	7	Historical and current information from this episode of care organized as a timeline	p. 3, lines 130–132; p. 4, lines 139–151	Reported in narrative form
Diagnostic Assessment	8a	Diagnostic testing, including physical examination, laboratory testing, imaging, and pathology	p. 4, lines 165–173	Reported
Diagnostic Assessment	8b	Diagnostic challenges	p. 4, lines 152–164; p. 20, lines 415–439	Reported; difficulty in identifying the primary site is described
Diagnostic Assessment	8c	Diagnosis, including other diagnoses considered	p. 4, lines 139–151, Table 1	Reported
Diagnostic Assessment	8d	Prognosis, where applicable	p. 5, lines 186–192	Reported
Therapeutic Intervention	9a	Types of therapeutic intervention	p. 4, lines 174–p. 5,185	Reported
Therapeutic Intervention	9b	Administration of therapeutic intervention, including dosage, strength, and duration	p. 4, lines 174–p. 5,185	Partly reported; treatment regimens and duration are described
Therapeutic Intervention	9c	Changes in therapeutic intervention with rationale	p. 5, lines 181–185	Reported
Follow-up and Outcomes	10a	Clinician- and patient-assessed outcomes, if available	Not applicable	Not available
Follow-up and Outcomes	10b	Important follow-up diagnostic and other test results	p. 5, lines 186–192	Reported
Follow-up and Outcomes	10c	Intervention adherence and tolerability	Not applicable	Partly reported
Follow-up and Outcomes	10d	Adverse and unanticipated events	p. 5, lines 186–192	Reported
Discussion	11a	A scientific discussion of the strengths and limitations associated with this case report	Discussion section and Limitations section	Reported
Discussion	11b	Discussion of the relevant medical literature with references	p. 19, lines 348–369 and following discussion text	Reported
Discussion	11c	The scientific rationale for the conclusions, including assessment of possible causes	p. 19, lines 371–387 and discussion thereafter	Reported
Discussion	11d	The primary take-away lessons of this case report in one-paragraph conclusion	Conclusion section	Reported
Patient Perspective	12	The patient’s perspective on the treatment(s) received	Not reported	Not available

Informed Consent	13	Did the patient give informed consent?	Yes	Reported

Abbreviations: CARE, CAse REport guidelines.

Note: Line numbers refer to the peer-review manuscript version submitted with this checklist. Items not applicable or not available are indicated accordingly. The checklist structure follows the CARE Checklist of Information to Include When Writing a Case Report.
